# A Rare Case of Enterococcus faecalis Keratitis in a Neurotrophic Cornea Successfully Treated With Topical Antibiotics and Amniotic Membrane

**DOI:** 10.7759/cureus.62463

**Published:** 2024-06-16

**Authors:** Magdalena Niestrata, Mohammad Saleki, Zahra Ashena

**Affiliations:** 1 Ophthalmology, Barking, Havering and Redbridge University Hospitals NHS Trust, London, GBR

**Keywords:** microbial keratitis, amniotic membrane, keratitis, enterococcus faecalis (e. faecalis), cornea

## Abstract

A patient in his 60s presented with severe keratitis in his right eye. He had a background of diabetes, high body mass index, arthritis and limited mobility, and high alcohol intake. Examination showed lower lid tarsal ectropion, floppy eyelid syndrome, advanced meibomian gland dysfunction, moderate neurotrophia, and large inferior keratitis with hypopyon. Corneal scrapes revealed *Enterococcus faecalis*, sensitive to vancomycin and ciprofloxacin only. Due to poor compliance with vancomycin, he was started on topical ciprofloxacin resulting in partial improvement but a persistent epithelial defect. Inserting a dry patch of amniotic membrane on the cornea accelerated epithelialization, and 11 weeks from presentation, complete corneal healing was noted.

In the presence of multiple systemic and ocular risk factors like diabetes, high body mass index, high alcohol intake, tarsal ectropion, floppy eyelid syndrome, neurotrophic cornea, blepharitis, and ocular surface inflammation, atypical keratitis, like this rare infection, should be suspected. The use of dry amniotic membrane has a role in epithelial healing in patients with neurotrophia.

## Introduction

Enterococci, gram-positive facultative anaerobes mainly found in the alimentary tract, typically cause various infections but are seldom associated with ocular issues [1]. While *Enterococcus*-induced ocular infections are rare, they have been reported in conditions such as post-cataract extraction endophthalmitis [2-4], conjunctivitis, and orbital cellulitis [5]. *Enterococcus faecalis* keratitis is an exceptionally uncommon infection, with previously reported cases shown in the Appendices [1,6-11]. Predominantly affecting females, *Enterococcus*-related keratitis is often linked to ocular surface diseases, corneal graft history, contact lens wearing, and steroid use [11]. This infection poses a clinical challenge due to its potential virulence, including reported cases of perforation at presentation, and the multi-antibiotic resistance of *Enterococcus* [11]. Although vancomycin is often effective, its unavailability for keratitis management, resistance, and potential epithelial toxicity present additional hurdles [6,9,11,12]. This study aims to contribute by reporting a case of *Enterococcus faecalis* keratitis in the background of moderate neurotrophia, shedding light on the associated clinical challenges.

## Case presentation

**Figure 1:**
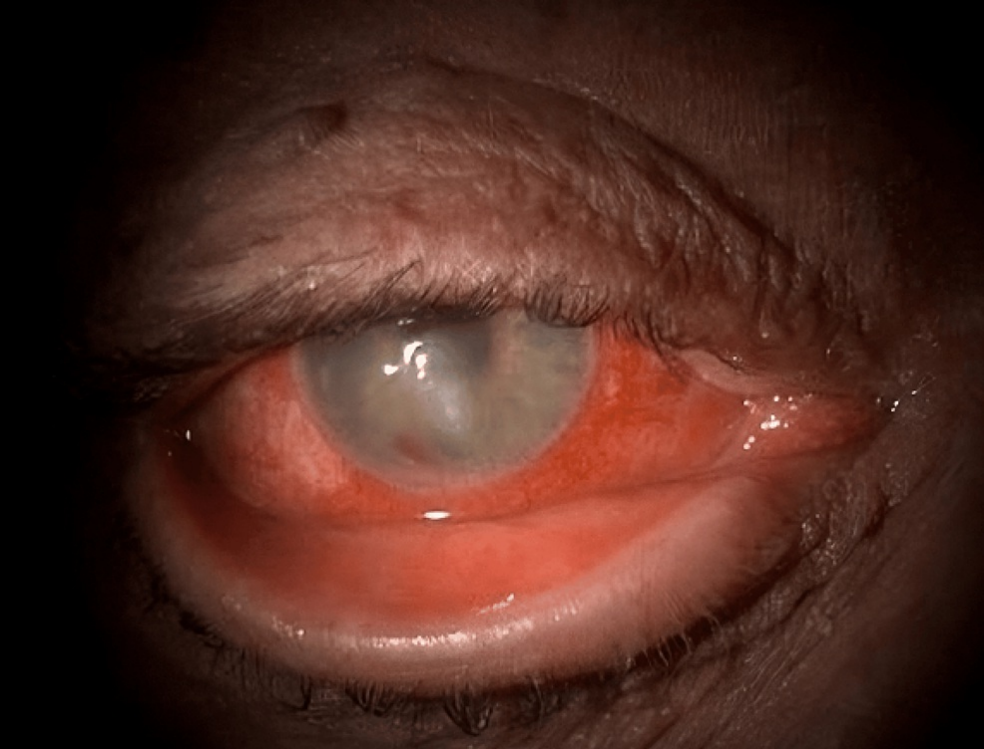
Right eye tarsal ectropion and corneal ulcer associated with hypopyon.

**Figure 2:**
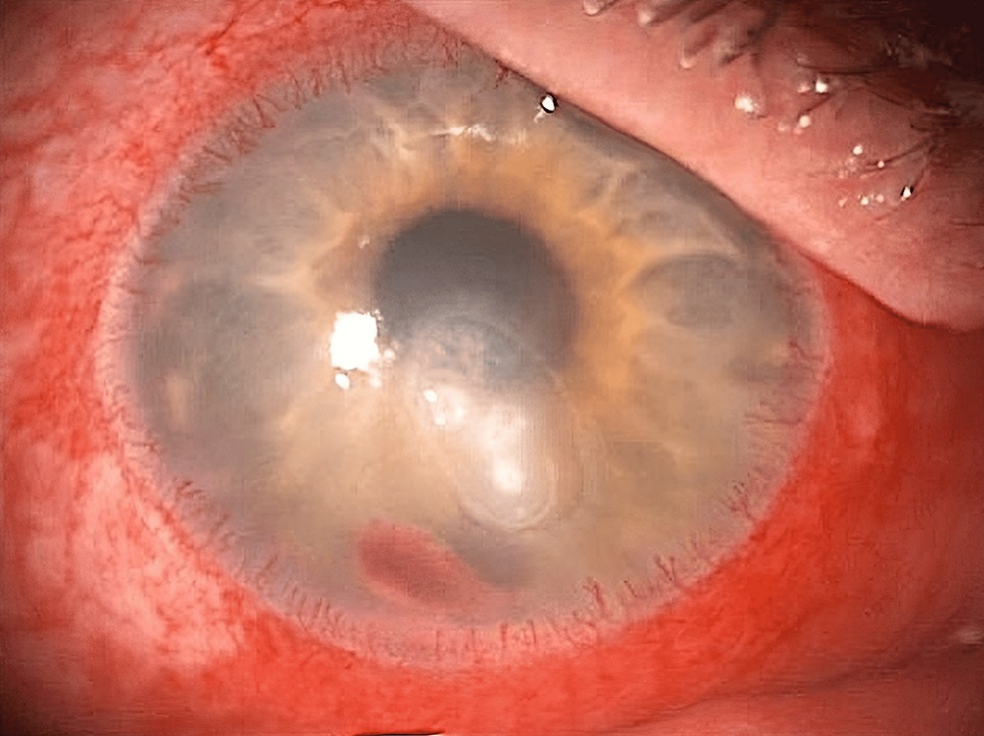
Inferior keratitis, hypopyon, and peripheral neovascularisation.

A man in his early 60s presented with painful redness and reduced vision in his right eye for two days. His left eye, densely amblyopic, relied on his right eye with a historic uncorrected visual acuity of 0.1 logMAR. He had a medical history of type II diabetes, high body mass index (BMI), and arthritis and a social history of a recent reduction in alcohol intake from 11 units to 4 units daily while living with his wife and dog. At presentation, his vision was 0.95 logMAR in the right eye and 1.1 logMAR in the left. Examination revealed a right lower lid tarsal ectropion, injected conjunctival vessels, large inferior keratitis (4.3 mm × 3.1 mm), and a 1.2 mm hypopyon (Figure 1 and Figure 2). Corneal sensation was partially reduced in both eyes, and the posterior segment view was limited, with no vitritis detected on the ultrasound scan. The ulcer did not look like a geographical ulcer, there was no localised neovascularisation, and he had no previous history of herpes simplex virus (HSV) keratitis.

Initial corneal scrapes were inconclusive, showing no organism growth on various agar types. A polymerase chain reaction (PCR) test for *Acanthamoeba* was negative. Treatment with levofloxacin yielded no improvement. A subsequent corneal scrape grew *Enterococcus faecalis*, sensitive to vancomycin, amoxicillin, and teicoplanin.

The patient initially received off-license topical vancomycin 5% eye drops hourly for 48 hours, causing discomfort and poor compliance due to burning sensations. Amoxicillin and teicoplanin were not available in the form of eye drops; therefore, sensitivity testing was repeated with available topical antibiotics, revealing sensitivity to ciprofloxacin. Hence, a switch to Ciloxan® 0.3% eye drops was prompted. This eye drop was applied every hour for 48 hours, followed by a daytime hourly application for another two days, and then reduced to a two-hour daytime application for another 10 days. Two weeks from commencing the intensive topical antibiotic, there was a partial improvement; however, the epithelial defect remained the same. Meibomian gland dysfunction, ocular surface inflammation, and moderate neurotrophic factors delayed healing. A tapering dose of preservative-free dexamethasone 0.1% was added thrice daily followed by twice and then once daily over three weeks, addressing discomfort and inflammation. During this time, the patient continued with ciprofloxacin eye drops six times a day.

**Figure 3:**
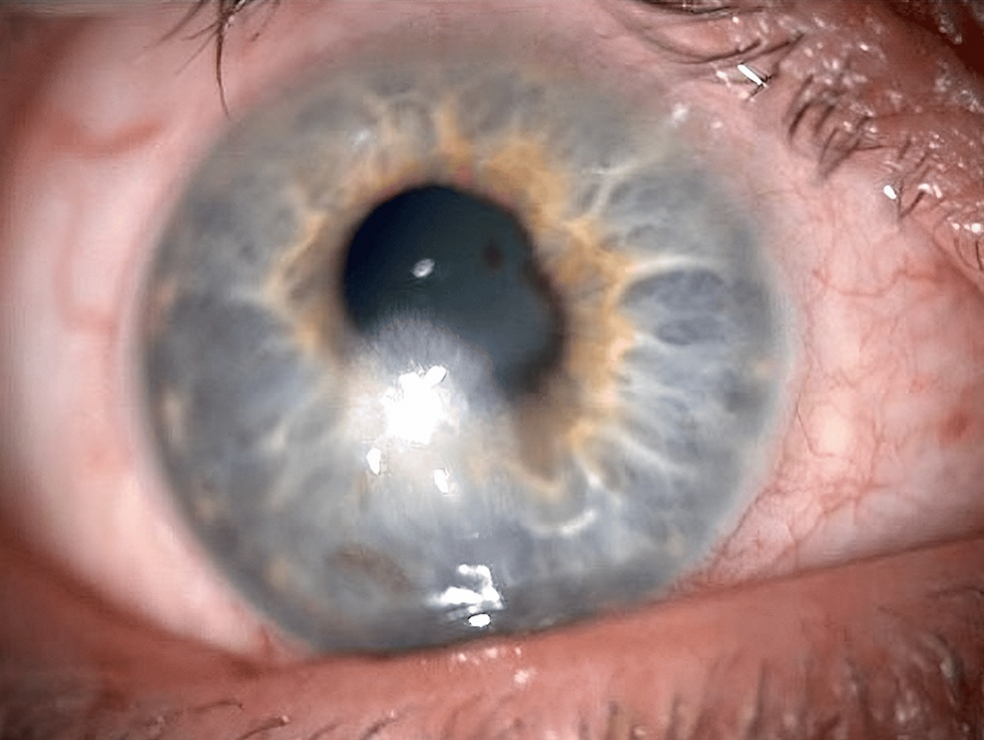
Persistent epithelial defect despite topical antibiotics and addressing the ocular surface inflammation.

**Figure 4:**
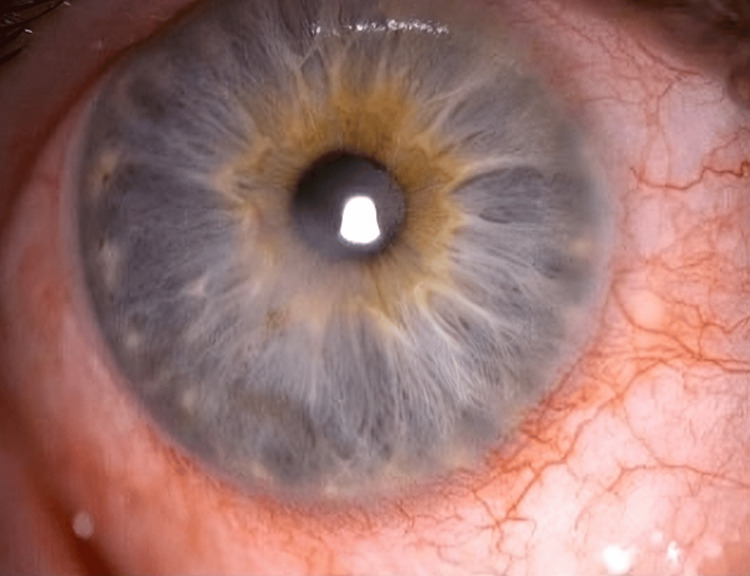
Resolved keratitis and fully healed epithelial defect 11 weeks from the presentation.

Five weeks from the beginning of the treatment, the epithelial defect measured 3.1 mm × 2.2 mm (Figure 3). At this point, amniotic membrane application initiated healing, reducing the defect to 1.5 mm × 0.7 mm after one week. The dry amniotic membrane disc was secured with a bandage contact lens, and to allow reasonable vision, it had a hole in the centre, which spared the visual axis. After the removal of the bandage contact lens, treatment continued with ciprofloxacin six times daily, preservative-free lubricating eye drops, nighttime lubricating ointment, lid hygiene, and once-daily preservative-free hydrocortisone sodium phosphate eye drop (Softacort®) for a month. Complete epithelialization, improved ocular surface (Figure 4), and unaided visual acuity of 0.1 logMAR were achieved 11 weeks post-presentation.

## Discussion

*Enterococcus faecalis* keratitis is an exceedingly rare occurrence, documented in only a number of cases prior to this report [1,6-11]. The typical source of this infection involves post-operative complications, often following keratoplasties [7,8] or as a result of ocular surgery-associated factors [1]. However, in this instance, the patient had no history of ocular surgery or contact lens use. Instead, his compromised ocular surface, secondary to meibomian gland dysfunction, tarsal ectropion, floppy eyelid syndrome, and neurotrophic cornea, possibly linked to diabetes and high alcohol intake, created an environment conducive to this rare infection and a non-healing epithelial defect. Given the initial corneal scrap was inconclusive, it could be assumed that this case was a viral keratitis with a secondary *Enterococcus faecalis* infection. However, primary bacterial infection remained suspected for a number of reasons including no previous history of HSV keratitis, round and well-demarcated edges of persistent epithelial defects (PED), rather than the geographical pattern in viral keratitis, absence of localised neovascularisation despite the chronicity of the PED, and equal neurotrophia in both cornea. Therefore, this was presumed to be a case of primary *Enterococcus faecalis* keratitis. Notably, the patient's interaction with a dog could have introduced *Enterococcus faecalis*, as multidrug-resistant strains have been identified in the intestinal tracts of animals [9].

*Enterococcus faecalis* poses a significant threat to the cornea, triggering an intense and rapidly progressing inflammatory response [7], often leading to necrosis and corneal melting [9], proving effective prompt treatment to be essential. Treating *Enterococcus faecalis* however presents considerable challenges. In vitro antibiotic sensitivity testing may not align with the clinical in vivo response to antimicrobials [6]. Although vancomycin is a common choice for sensitivity, its usage is limited by factors such as ocular toxicity and poor patient compliance [6,8,9,11]. Additionally, the scarcity of topical vancomycin in the UK, compounded by its off-label use, introduces delays in treatment initiation. The rise of intrinsic and acquired resistance to multiple antibiotics, including vancomycin, further complicates the therapeutic landscape.

Alternative treatments have shown promise in vancomycin-resistant cases. Various topical antibiotic regimens have been explored, with combinations like fortified vancomycin and ciprofloxacin proving effective [11]. In cases refractory to monotherapy, combinations like fortified tobramycin and cefazolin [11] or vancomycin and gentamicin [7] have been successful.

In this reported case, despite topical treatment, an epithelial defect persisted. The management involved the application of a dry amniotic membrane, leveraging its regenerative properties. To our knowledge, this is the first reported case of dry amniotic membrane use in an *Enterococcus faecalis* infection and neurotrophic cornea. Amniotic membrane, whether dehydrated or not, has proven effective in treating PED and non-healing corneal ulcers [13]. This has been shown to have a 70% success rate in corneal ulcers and established as useful in a variety of aetiologies such as herpetic ulcers, rheumatic disease, penetrating keratoplasty, and trauma [14]. Examples of infection-related epithelial defects treated with amniotic membrane include *Pseudomonas*, *Acanthamoeba*, and *Aspergillus *keratitis [15]. In this instance, the patient experienced improvement despite retaining the membrane for only six days, highlighting the potential benefit of amniotic membrane use for PED in *Enterococcus faecalis* infection, even in limited application.

While the patient continued to recover, further corrective measures, including surgical intervention for ectropion correction, were considered. Two years following the management of keratitis and optimising his ocular surface, there has been no recurrence of keratitis. This multifaceted case emphasizes the intricate challenges in diagnosing and treating *Enterococcus faecalis* keratitis, showcasing the need for tailored approaches and continued exploration of alternative treatments such as amniotic membrane application.

## Conclusions

*Enterococcus faecalis *keratitis may be suspected when multiple ocular and systemic risk factors are present. This rare infection presents a clinical challenge due to its high virulence, multi-antibiotic resistance, and poor antibiotic compliance. Managing such complex cases requires a staged approach, focusing on treating the infection and addressing all comorbidities to achieve success. This case also highlights the benefit of using a dry amniotic membrane to aid in the healing of PED in *Enterococcus faecalis* keratitis with moderate neurotrophia.

## Appendices

**Table 1:** Summary of the reported cases with Enterococcus faecalis keratitis LP: light perception; IOL: intra-ocular lens; NA: not applicable; CF: counting fingers; PKP: penetrating keratoplasty; BCL: bandage contact lens; DMEK: Descemet membrane endothelial keratoplasty; DSEK: Descemet's stripping endothelial keratoplasty; SCL: scleral contact lens; AF: atrial fibrillation; CCF: congestive cardiac failure; CVA: cerebrovascular attack; AIDS: acquired immune deficiency syndromes

Case	Age (y)	Sex	Presenting visual acuity	Social history	Past medical history	Contact lens wearer	Past ocular history	Topical drops	Outcome	Culture results	Polymicrobial
Lee and Lee, 2004 [1]	67	F	LP	NA	Diabetes mellitus	No	Phaco/IOL, endophthalmitis	None	Intravitreal vancomycin, amikacin, and amphotericin; second intravitreal injection on vancomycin, ceftazidime, and dexamethasone; third intravitreal injection of vancomycin and dexamethasone; topical vancomycin and fortified amikacin. Vitreous opacity resolved but corneal opacity and neovascularisation remained	E. faecalis	No
Subashini et al., 2007 [6]	30	F	CF	NA	NA	No	Injury to the eye with fingernail	Ofloxacin	Resolved with a remaining corneal scar with topical vancomycin	E. faecalis	No
Sherman et al., 1992 [7]	61	M	20/400	NA	NA	BCL	PKP, herpetic keratouveitis	None	Resolved on vancomycin and gentamicin	E. faecalis	No
Sudana et al., 2021 [8]	64	M	20/600	NA	NA	No	DMEK, pseudophakia	Presumed topical steroids (post-op graft patient but steroids not specifically mentioned in report)	Topical vancomycin, removal of DMEK, new DSEK	E. faecalis	No
Peng et al., 2009 [9]	23	F	20/50	Works as a pet groomer	NA	No	No	None	Cefazolin sodium, amikacin, ciprofloxacin, vancomycin, epithelial debridement	E. faecalis	No
Lam et al., 1993 [10]	65	F	NA	NA	NA	BCL	PKP, endophthalmitis, alkali burns, cataract extraction, cyclocryotherapy for glaucoma	None	Fortified vancomycin, PKP	E. faecalis	No
Lam et al., 1993 [10]	88	F	CF	NA	NA	No	PKP, amblyopia	None	Fortified vancomycin, PKP, extracapsular cataract extraction, IOL implantation	E. faecalis	No
Rau et al., 2008 [11]	17	F	20/200	NA	None	Yes, SCL	None	None	Resolved on moxifloxacin	*B. cereus*, *E. faecalis*, *K. pneumoniae*	Yes
Rau et al., 2008 [11]	21	F	20/40	NA	None	Yes, SCL	None	None	Resolved on moxifloxacin	*P. aeruginosa*, *K. pneumoniae*, *E. faecalis*	Yes
Rau et al., 2008 [11]	85	M	HM	NA	Anemia, hypothyroidism, hypertension	No	Lamellar keratoplasty, two PKP, Mooren ulcer	Cyclosporine (2/2), artificial tears, tobramycin/ dexamethasone	PKP #3	E. faecalis	No
Rau et al., 2008 [11]	58	F	20/25	NA	None	Yes, SCL	Epithelial basement membrane dystrophy	Artificial tears	Resolved on moxifloxacin	*E. faecalis* Coagulase-negative *S. aureus*	Yes
Rau et al., 2008 [11]	55	F	NA	NA	NA	Yes, SCL	None	NA	PKP	E. faecalis	No
Rau et al., 2008 [11]	73	M	NA	NA	Bladder cancer	No	PKP	NA	NA	E. faecalis	No
Rau et al., 2008 [11]	57	F	NA	NA	None	Yes, SCL	None	None	Resolved on fortified vancomycin	E. faecalis	No
Rau et al., 2008 [11]	89	F	NA	NA	None	No	AMD	None	Resolved on fortified vancomycin	E. faecalis	No
Rau et al., 2008 [11]	23	F	20/60	NA	None	Yes, SCL	None	None	Resolved on fortified vancomycin, ciprofloxacin	*P. aeruginosa*, *E. faecalis*, *S. marcescens*	Yes
Rau et al., 2008 [11]	85	F	5/200	NA	Hypercholesterolemia	Yes, SCL	Cataract extraction	Timoptic 0.5%	Resolved on ofloxacin	E. faecalis	No
Rau et al., 2008 [11]	41	F	CF	NA	None	No	Entropion	None	Resolved on moxifloxacin	*E. faecalis*, *S. aureus*	Yes
Rau et al., 2008 [11]	49	M	20/30	NA	AIDS	No	Foreign body removal	None	Resolved on ofloxacin	E. faecalis	No
Rau et al., 2008 [11]	98	F	CF	NA	AF, CCF, CVA, ovarian cancer, anemia, pacemaker	No	Entropion	None	PKP	E. faecalis	No
Rau et al., 2008 [11]	74	F	20/300	NA	Schizophrenia, depression, paranoia, osteoporosis, hypothyroidism	No	Grave's ophthalmopathy, lagophthalmos	Pred Forte, Polytrim, Polysporin	Resolved on ciprofloxacin	*E. faecalis*, *S. sciuri* *Corynebacterium* species	Yes
Rau et al., 2008 [11]	37	F	CF	NA	Migraine, appendectomy	Yes, SCL	Keratoconus	None	Resolved on fortified tobramycin and cefazolin	E. faecalis	No
